# A Feature Selection Method Based on an Improved Sand Cat Swarm Optimization Algorithm with Multi-Strategy Fusion

**DOI:** 10.3390/e28060595

**Published:** 2026-05-26

**Authors:** Zhouheng Wu, Tao Zhou, Jianyong Fan, Ruimin Zhang, Zhigang Li, Kang Hu

**Affiliations:** 1 College of Information Science and Technology, Shihezi University, Shihezi 832003, China; 20242108025@stu.shzu.edu.cn (Z.W.); hukang@stu.shzu.edu.cn (K.H.); 2 Xinjiang Electronic Research Institute Co., Ltd., Urumqi 830000, China; fanjy_dzs@163.com; 3 College of Mechanical and Electrical Engineering, Shihezi University, Shihezi 832003, China; zrm_inf@shzu.edu.cn

**Keywords:** feature selection, SCSO algorithm, multi-strategy fusion, high-dimensional data

## Abstract

Feature selection (FS) plays a crucial role in high-dimensional data analysis by improving model performance and reducing computational complexity. However, existing metaheuristic-based FS methods often suffer from insufficient population diversity, premature convergence, and limited capability to escape local optima, which substantially constrains their effectiveness in complex search spaces. To address these challenges, this paper proposes a novel Improved Sand Cat Swarm Optimization algorithm with multi-strategy fusion (ISCSO) for feature selection. The proposed method introduces a hybrid initialization mechanism based on the Hénon chaotic map and lens imaging reverse learning to enhance population diversity. A golden sine-based phase adjustment strategy is further incorporated to achieve a more effective balance between global exploration and local exploitation. In addition, a nonlinear adaptive weight mechanism is designed to dynamically regulate the search process, while a simulated annealing-based acceptance criterion is integrated to improve the ability to escape local optima. Comprehensive experiments are conducted on the CEC2017 benchmark suite and 18 real-world datasets from the UCI repository. The results demonstrate that ISCSO achieves superior performance over state-of-the-art algorithms, obtaining the optimal results on 82.76% of benchmark functions. In feature selection tasks, ISCSO achieves the optimal average fitness on 94.44% of datasets, reduces feature dimensionality significantly, and consistently improves classification accuracy. These findings indicate that ISCSO provides a competitive and reliable solution for high-dimensional feature selection and complex optimization problems.

## 1. Research Background

### 1.1. The Importance of Feature Selection in High-Dimensional Data Processing

**Figure 1:**
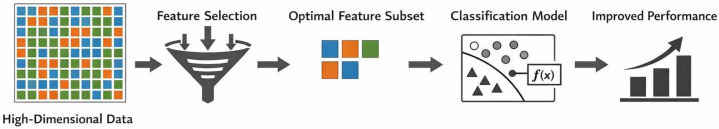
Optimization process of feature selection.

As a key preprocessing step in high-dimensional data processing, feature selection aims to eliminate redundant and irrelevant features by selecting a minimal optimal feature subset, thereby alleviating the curse of dimensionality and improving classifier performance [1]. In the era of explosive development of information technology, high-dimensional data has become a core research object in fields such as bioinformatics [2], medical image analysis [3,4], and financial risk prediction [5]. For example, a single sample of gene microarray data typically contains 10,000–50,000 gene expression features, yet less than 1% of these features have significant biological significance [6]. This “curse of dimensionality” poses severe challenges to traditional machine learning models [7], including exponentially growing computational complexity due to redundant features, increased risk of overfitting from irrelevant features, and lack of interpretability arising from nonlinear correlations between features [8]. Feature selection technology can significantly reduce computational resource consumption [9] while maintaining or improving classification performance. Studies have shown that the rational application of feature selection in diagnostic classification tasks for specific cancers improves model performance and generalization capability while reducing costs [10]. The feature selection process is shown in Figure 1.

### 1.2. Application Status of Swarm Intelligence Algorithms in Feature Selection

Traditional feature selection methods are mainly categorized into three types: Filter, Wrapper, and Embedded [11]. Among them, Wrapper methods have attracted significant attention due to their direct optimization of classification performance. However, when the feature dimension exceeds 1000, the complexity of exhaustive search reaches O($2^{n}$) [12], placing traditional deterministic algorithms in a computational dilemma. Swarm intelligence algorithms provide a new solution for high-dimensional feature selection by simulating the collaborative behavior of biological groups [13]. Typical examples include the following: Genetic Algorithm (GA) [14]: Achieves global search through crossover and mutation operators which often suffer from premature convergence. Particle Swarm Optimization (PSO) [15]: Updates positions using individual and group experience which is sensitive to the curse of dimensionality in high-dimensional spaces. Grey Wolf Optimization (GWO) [16]: Employs a hierarchy-based position update mechanism but lacks sufficient local exploitation capability.

### 1.3. Motivation and Contributions

Sand Cat Swarm Optimization (SCSO) [17], as a new type of swarm intelligence algorithm, realizes optimized search by simulating the hunting behavior of sand cats. Although the original SCSO has demonstrated promising performance in continuous optimization problems, its application to feature selection, especially in high-dimensional and small-sample datasets, faces four critical limitations: Poor population diversity: SCSO’s random initialization often leads to uneven distribution of individuals in the search space, which is exacerbated in high-dimensional spaces, resulting in the algorithm being trapped in local optima before exploring potential optimal feature subsets [18]. Rigid position update mechanism [19]: The fixed linear convergence factor G fails to adapt to the non-uniform distribution of feature spaces, reducing convergence speed in high-dimensional data. Lack of local optima escape capability [20]: SCSO lacks an explicit mechanism to accept inferior solutions, making it difficult to jump out of local optimal solutions in rugged fitness landscapes caused by high-dimensional feature redundancy and small-sample noise. Mismatched weight strategy: Constant coefficients cannot dynamically adjust the search direction, resulting in unstable classification performance.

To overcome these limitations, this paper proposes an Improved Sand Cat Swarm Optimization algorithm (ISCSO) with multi-strategy fusion. The main contributions of this study are summarized as follows: 1. A chaotic initialization strategy based on the Hénon map and lens reverse learning is introduced to enhance population diversity. 2. A golden sine-based phase adjustment mechanism is incorporated to improve the balance between exploration and exploitation. 3. A nonlinear adaptive weight strategy is designed to dynamically adjust the search process. 4. A simulated annealing mechanism is integrated to enhance the ability to escape local optima. The core innovation of ISCSO lies in the systematic and synergistic fusion of the above four complementary strategies, each targeting a specific defect of the original SCSO. This integrated design significantly outperforms single-strategy improvements and enables strong performance in high-dimensional feature selection.

In particular, the proposed method is well suited for parameter tuning in image processing tasks for defect detection [21]. Traditional defect detection algorithms (e.g., Canny edge detection, morphological operations, and threshold segmentation) rely on manual experience for parameter adjustment [22].

## 2. Related Work

### 2.1. Sand Cat Swarm Optimization Algorithm

The sand cat is a mammal that lives in harsh environments such as sandy and stony deserts in Central Asia’s Sahara and the Arabian Peninsula. The hunting mechanism of sand cats is particularly distinctive. They use their acute hearing ability to detect low-frequency noises, through which they can find and prey on animals moving underground. Studies have shown that sand cats have a strong ability to receive sounds below 2 kHz. These unique characteristics may be the reason why sand cats can detect noises, track prey, and successfully attack based on the prey’s position. The Sand Cat Swarm Optimization algorithm simulates the behavior of sand cats during hunting, which is divided into two phases: exploration and predation (exploitation). The mathematical model is as follows:

In the search phase, sand cats update their positions according to the optimal solution, their current position, and the sensitivity range to search for other possible optimal prey positions, which can find new local optima in new search areas. The obtained position is between the current position and the prey position, and at the same time, randomness ensures a low operating cost and complexity of the algorithm. The mathematical modeling of the above search process is as follows:

(1) $$\vec{\mathrm{Pos}}\left(t+1\right)=\vec{r}\cdot \left(\vec{{\mathrm{Pos}}_{bc}}\left(t\right)-\mathrm{rand}\left(0,1\right)\cdot \vec{{\mathrm{Pos}}_{c}}\left(t\right)\right)$$

(2) $$\vec{r}=\vec{r_{G}}\times \mathrm{rand}\left(0,1\right)$$

(3) $$\vec{r_{G}}=s_{M}-\left(\frac{s_{M}\times {\mathrm{iter}}_{c}}{{\mathrm{iter}}_{\max}}\right)$$

In the formula, $Pos_{bc}$ is the optimal solution, $Pos_{c}$ is the current position, and $\vec{r}$ is the sensitivity range of the sand cat individual, used in the position update formula of the sand cat individual, which is generated by $\vec{r_{G}}$ multiplying by a random number, as shown in Equation (2). $\vec{r_{G}}$ refers to a dynamically adjusted parameter, representing the global sensitivity range of the sand cat individual during the search process, which is calculated by Equation (3). $s_{M}$ is the maximum sensitivity range of the sand cat (the default value is 2, corresponding to the 2 kHz low-frequency detection ability of the sand cat mentioned above). ${\mathrm{iter}}_{c}$ is the current number of iterations, and ${\mathrm{iter}}_{\max}$ is the maximum number of iterations. In the exploration phase, $\vec{r_{G}}$ is relatively large (close to $S_{M}=2$), and sand cat individuals search the global space randomly with large step sizes to avoid falling into local optima.

After locating underground prey according to the ability of their ears, sand cats adjust their body direction and dig towards the prey. The position update formula can be expressed as

(4) $$\vec{\mathrm{Pos}}\left(t+1\right)=\vec{{\mathrm{Pos}}_{b}}\left(t\right)-\vec{r}\cdot \vec{{\mathrm{Pos}}_{\mathrm{md}}}\cdot \cos\left(\theta \right)$$

(5) $$\vec{{\mathrm{Pos}}_{\mathrm{rnd}}}=\left|\mathrm{rand}\left(0,1\right)\cdot \vec{{\mathrm{Pos}}_{b}}\left(t\right)-\vec{{\mathrm{Pos}}_{c}}\left(t\right)\right|$$

$\vec{{\mathrm{Pos}}_{\mathrm{rnd}}}$ represents the random distance between the current position $\vec{{\mathrm{Pos}}_{c}}$ and the optimal solution $\vec{{\mathrm{Pos}}_{b}}$, which is calculated by Equation (5). This distance reflects the gap between the sand cat and the optimal solution, providing a basis for subsequent actions. The sensitivity range of the sand cat is regarded as a circle, and the roulette wheel selection algorithm is used to select a random angle (θ) between 0 and 360 degrees to determine the moving direction. Since the selected angle value is between −1 and 1, each individual can move in different directions in the search space to avoid falling into local optima.

(6) $$R=2\times \vec{r_{G}}\times \mathrm{rand}\left(0,1\right)-\vec{r_{G}}$$

The sand cat optimization algorithm achieves seamless switching between exploration and exploitation phases via the dynamic adjustment of parameters *R* and $\vec{r_{G}}$. The calculation formula of *R* is Equation (6). When |R| > 1, the sand cat is in the exploration phase; when |R| ≤ 1, the sand cat is in the predation (exploitation) phase. This mechanism imitates the behavior of sand cats from large-scale search to precise hunting.

### 2.2. Hénon Chaotic Map

The Hénon map is a classical two-dimensional discrete chaotic system introduced by the French astronomer Michel Hénon, and is commonly employed to study the chaotic behavior of nonlinear dynamical systems. Its core idea is to generate complex chaotic trajectories through simple nonlinear iterative equations, and it has the characteristics of being sensitive to initial conditions and having unpredictable long-term behavior [23]. Its iterative equation is shown in Equation (7):

(7) $$\left\{\begin{array}{c} x_{n+1}=1+y_{n}-ax_{n}^{2}\\ y_{n+1}=bx_{n} \end{array}\right.$$

In the equation, *a* and *b* are control parameters, which are usually set to *a* = 1.4 and *b* = 0.3, respectively. At this time, the system exhibits chaotic behavior. *a* dominates the nonlinear term and determines the complexity of the trajectory; *b* controls the lateral contraction and affects the shape of the attractor.

### 2.3. Golden Sine Algorithm

The Golden Sine Algorithm is a metaheuristic optimization algorithm proposed by Tanyildizi et al. in 2017. It is inspired by the scanning of the sine function in the unit circle [24], which is similar to the search of the solution space of the optimization problem. At the same time, it combines the golden section ratio to narrow the search space to approach the optimal solution. The algorithm introduces the golden section coefficients $x_{1}$ and $x_{2}$ in the position update to balance the “search” (global search to find potential regions) and “exploitation” (local search to refine the optimal solution). The calculation formula is shown in Equation (8).

(8) $$\left\{\begin{array}{c} x_{1}=a+b\left(1-\tau \right)\\ x_{2}=a\left(1-\tau \right)+b\tau \end{array}\right.$$

In the equation, $\tau =\frac{\sqrt{5}-1}{2}$ is the golden section ratio, *a* and *b* are the initial values of the golden section ratio search, which are −π and π by default. The position of the search agent is updated through a specific formula to make it continuously approach the optimal solution, and the formula is shown in Equation (9).

(9) $$P_{i}\left(t+1\right)=P_{i}^{t}\left|\sin r_{1}\right|-r_{2}\sin r_{1}\left|x_{1}D^{b}-x_{2}P_{i}^{t}\right|$$

In the equation, $r_{1}$ and $r_{2}$ are random numbers. $r_{1}$ ∈ [0, 2π] determines the moving distance of the iterative individual; $r_{2}$ ∈ [0, π] determines the position update direction of the iterative individual.

### 2.4. Nonlinear Adaptive Weight

Adaptive weight refers to dynamically adjusting the value of the weight according to the characteristics of the problem during the iteration of the algorithm to balance the global search and local optimization capabilities [25]. This paper proposes a nonlinear adaptive weight method. The algorithm uses a higher weight in the initial stage to achieve stronger global search performance and ensure a wide search range. As the number of iterations increases, when approaching the optimal solution, the weight value will decrease exponentially, thereby significantly enhancing the local search ability of the algorithm. Moreover, using nonlinear adaptive weights can better balance global and local search capabilities and adapt to complex situations more flexibly. The adaptive weight formula is shown in Equation (10).

(10) $$w\left(t\right)=w_{\max}-\left(w_{\max}-w_{\min}\right)\cdot {\left(\frac{t}{T}\right)}^{k}+\sigma \cdot N\left(0,1\right)$$

Among them, $w_{\max}$ and $w_{\min}$ are the initial maximum and minimum weights, which are usually set to 0.9 and 0.4, respectively. *t* and *T* are the current number of iterations and the total number of iterations, respectively. *k* and σ are the attenuation coefficient and Gaussian perturbation intensity, which are usually set to 2 and 0.1. N(0,1) is a random number that conforms to the standard normal distribution [26].

### 2.5. Simulated Annealing Algorithm

The simulated annealing algorithm is a stochastic optimization algorithm based on the Monte Carlo iterative solution strategy. It is derived from the annealing process of solid materials [27]. The solid is heated to a high temperature and then slowly cooled. The internal particles gradually change from a disordered state to an ordered state, and finally reach a stable state with the lowest energy at room temperature. In the algorithm, the internal energy is simulated as the objective function value, and the temperature evolves into a control parameter. Starting from a certain high initial temperature, with the continuous decrease in the temperature parameter, combined with the probability jump characteristics, the global optimal solution of the objective function is randomly searched in the solution space. That is, it allows for the jumping out of the local optimal solution with a certain probability and finally tends to the global optimal solution [28]. Its core mechanism is the Metropolis acceptance criterion, as shown in Equation (11), which is used to determine whether to accept a worse new solution.

(11) $$P\left(\Delta E,T\right)=\left\{\begin{array}{cc} 1 & \Delta E<0\\ \exp\left(-\frac{\Delta E}{T}\right) & \Delta E\geq 0 \end{array}\right.$$

(12) $$T_{k+1}=\alpha \cdot T_{k}$$

In the equation, ΔE is the difference between the objective function of the new solution and the current solution. *T* represents the current temperature, which is changed through different temperature update strategies. In this paper, exponential cooling is adopted, and the formula is shown in Equation (12).

## 3. Improved Sand Cat Swarm Optimization Algorithm with Multi-Strategy Fusion

To address the issues of low initialization quality, slow convergence speed, and a tendency to fall into local optima of the traditional SCSO algorithm in high-dimensional feature selection tasks, this paper integrates the four strategies mentioned above and proposes the ISCSO algorithm. The improved algorithm significantly improves the feature selection performance by dynamically balancing the global exploration and local exploitation capabilities. The modified formulas for the search phase (Equation (1)) and the exploitation phase (Equation (4)) become Equations (13) and (14).

(13) $$\vec{\mathrm{Pos}}\left(t+1\right)=w\cdot \vec{r}\cdot \left(\vec{{\mathrm{Pos}}_{bc}}\left(t\right)-\mathrm{rand}\left(0,1\right)\cdot \vec{{\mathrm{Pos}}_{c}}\left(t\right)\right)\cdot C+G$$

(14) $$\vec{\mathrm{Pos}}\left(t+1\right)=\vec{{\mathrm{Pos}}_{b}}\left(t\right)-w\cdot \vec{r}\cdot \vec{{\mathrm{Pos}}_{\mathrm{md}}}\cdot \cos\left(\theta \right)\cdot C+G$$

(15) $$C=\frac{x_{\mathrm{current}}-x_{\min}}{x_{\max}-x_{\min}}$$

(16) α=2π·rand(0,1)

(17) G=τ·sin(α)

Among them, *w* is the adaptive weight mentioned above. *C* represents the perturbation term based on the Hénon chaos, generated by the current chaos sequence value, and the generation formula is Equation (15); $x_{current}$ is the current Hénon sequence value. *G* is the golden sine term, and the calculation formulas are Equations (16) and (17). Finally, combined with the Metropolis criterion in the simulated annealing algorithm, worse solutions are accepted with a certain probability.

The original SCSO employs a uniform distribution to randomly generate the initial population, which tends to result in population aggregation and uneven coverage of the search space. The improved SCSO algorithm introduces initialization using the Hénon chaotic map, and generates a highly diverse initial population through the ergodicity and initial value sensitivity of the two-dimensional chaotic system, which significantly enhances the initialization diversity. Compared with Logistic and Tent maps, the Hénon map provides more complex chaotic sequences and stronger ergodicity, which helps to generate a more uniform initial population. This is more suitable for high-dimensional feature selection spaces where random initialization easily leads to aggregation.

The fixed decay mode of the original SCSO fails to adapt to complex search spaces, which leads to slow convergence in the later stage. We introduce the golden section coefficient and nonlinear adaptive weight from the golden sine algorithm, which greatly improves the convergence speed by dynamically adjusting the phase, optimizing the search direction, nonlinearly decaying the weight with the number of iterations, and balancing the exploration and exploitation phases. The golden sine strategy introduces the golden section ratio to adjust the search direction smoothly. Compared with random angle adjustment in the original SCSO, it improves the stability of convergence and avoids frequent oscillation. Compared with linear weights, nonlinear adaptive weights provide a stronger global search at the early stage and a finer local exploitation at the later stage, which better matches the dynamic requirements of feature selection.

The original SCSO lacks an effective escape mechanism, has poor adaptability to multimodal optimization problems, and the random search leads to high redundancy of feature subsets, which limits the performance of the classifier. This paper introduces the simulated annealing mechanism and chaotic perturbation term. By accepting inferior solutions through temperature decay and the Metropolis criterion, and introducing random perturbations using the ergodicity of Hénon chaos, the global search capability is significantly enhanced and key features can be accurately located. Compared with random mutation or restart strategies, the Metropolis criterion accepts inferior solutions in a controlled manner, which effectively prevents premature convergence without introducing excessive randomness or computational cost.

At the same time, the collaboration between the Hénon chaos and the simulated annealing algorithm combines determinism and randomness, enhances the stability of the results, and improves the robustness of the algorithm.

**Figure 2:**
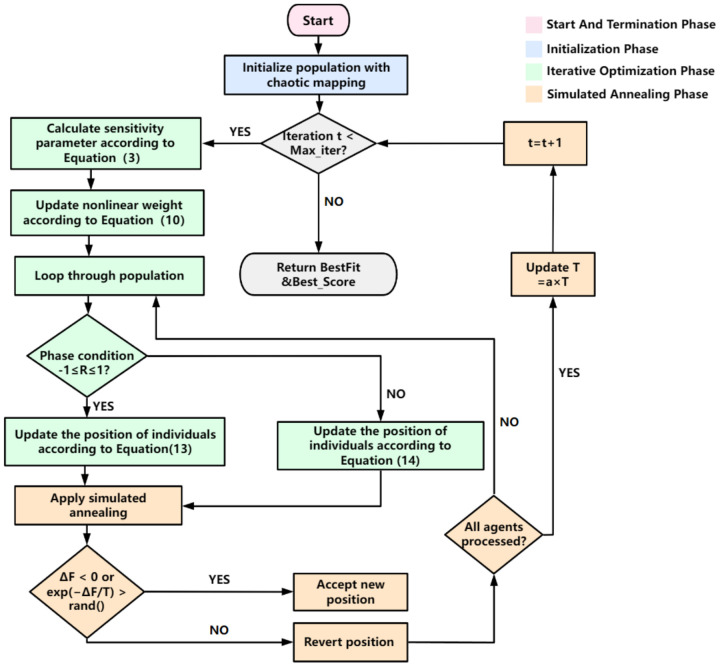
Flowchart of the ISCSO algorithm.

The overall flowchart of the algorithm is shown in Figure 2, and the overall steps of the algorithm are as follows in Algorithm 1:   
**Algorithm 1:** The pseudocode of the ISCSO algorithm**Require**: Max_iter (Maximum iterations), SearchAgents_no (Population size), lb/ub (Bounds)**Ensure**: BestFit (Optimal feature subset), Best_Score (Fitness value)1: Initialize population with chaotic mapping2: Fitness function *f*(*x*): Equation (21).3: **while** *t* < Max_iter **do**4:        Calculate sensitivity parameter according to Equation (3)5:        Update nonlinear weight according to Equation (10)6:        **for** each agent i in population **do**7:                 **if** (−1 ≤ R ≤ 1) **then**▸ Exploitation phase8:                         Update position using:9:                         according to Equation (13)10:                **else** ▸ Exploration phase11:                        Select random candidate CP12:                        Update position using:13:                        according to Equation (14)14:                **end if**15:                        Apply simulated annealing:16:                **if** Δ*F* < 0 **or** exp(−Δ*F*/*T*) > rand() **then**17:                        Accept new position18:                **else**19:                        Revert to previous position20:                 **end if**21:        **end for**22:        Update temperature *T* = $\alpha^{*}T$23:        *t* = *t*+ 124: **end while**25: **Return** BestFit, Best_Score

## 4. Experimental Design and Results Analysis

This study conducted two key experiments to test the effectiveness and accuracy of the proposed ISCSO method. The first experiment aims to evaluate the performance of the ISCSO algorithm on global optimization problems, using 29 functions from the CEC2017 test suite [29]. This suite covers a variety of optimization problems, including unimodal functions, multimodal functions, and fixed-dimension multimodal functions. These benchmark functions enable us to effectively assess the performance of the ISCSO algorithm in various environments. The second experiment is applied to feature selection, utilizing 18 classic datasets that span different domains and present varying difficulties in feature selection. By applying the ISCSO algorithm to these datasets, we can evaluate its performance in practical data processing scenarios [30]. As a crucial step in machine learning and data mining, feature selection significantly impacts model performance and complexity [31]. Thus, testing the ISCSO algorithm in feature selection tasks can effectively verify its practicality.

All experiments were conducted on the 64-bit version of MATLAB R2024a. The hardware configuration included an AMD Ryzen 7 4800H processor, an RTX 2060 graphics card, and 16 GB of memory. In both experiments, the results of the ISCSO algorithm were compared with those of the original SCSO algorithm and several other commonly used metaheuristic algorithms. Multiple performance metrics were employed to evaluate these algorithms, thereby comprehensively demonstrating the relative advantages of the ISCSO algorithm and its potential for future applications.

### 4.1. Performance Metrics of Experiments

To evaluate the performance of the Improved Sand Cat Swarm Optimization algorithm, the following evaluation metrics are used in this paper: Accuracy: It is used to directly reflect the correct rate of the algorithm in all predictions, and its calculation formula is given by Equation (18). Here, *C* is the number of correctly classified samples, and *N* is the total number of samples.

(18) $$acc=\frac{C}{N}$$

(19) $$acc_{m}=\frac{\sum_{i=1}^{R}acc_{i}}{R}$$

Average accuracy: It refers to the average value of the classification accuracy after *R* runs, and the calculation formula is Equation (19). Here, $acc_{i}$ is the accuracy of the *i* run.

(20) $$std=\sqrt{\frac{\sum_{l=1}^{R}{\left(x_{l}-x_{\mathrm{mean}}\right)}^{2}}{R}}$$

Standard deviation: This metric evaluates the consistency and stability of the optimization algorithm across multiple runs. A lower standard deviation indicates greater stability of the algorithm, and its calculation formula is Equation (20). Here, *R* is the number of independent runs; $x_{i}$ and $x_{\mathrm{mean}}$ represent the measured value and the average value in the *i*-th run, respectively.

(21) $$fitness=\alpha \times ER+\beta \times \frac{F_{seclect}}{F_{max}}$$

Fitness: Fitness jointly evaluates the classification error rate and the proportion of selected features relative to the total number of features. A lower fitness value indicates better performance, which means minimizing the number of feature selections while reducing the classification error rate to achieve the optimal effect. The formula for fitness is shown in Equation (21). Among them, let α=0.9, β=0.1, the total number of features be $F_{max}$, the number of selected features be $F_{select}$, and the classification error rate be ER=1−acc.

(22) $$fitness_{m}=\frac{\sum_{i=1}^{R}fitness_{i}}{R}$$

Average fitness: This is the average of the classification accuracy after R runs, and the calculation formula is Equation (22). In global optimization, the number of independent runs *R* is assigned the value of 30. In feature selection, *R* is assigned the value of 20. $fitness_{i}$ represents the fitness value obtained from the initial run.

(23) $$feature_{m}=\frac{\sum_{i=1}^{n}size\left(i\right)}{R}$$

Average number of selected features: Equation (23) represents the average number of features selected in *R* runs, where size(i) represents the number of features selected in the *i*-th run.

### 4.2. Algorithm Parameter Settings

The parameter settings have a significant impact on algorithm efficiency and experimental results, directly affecting the search capability, convergence speed, and final performance of the algorithm. Table 1 shows the parameter configurations of the Improved Sand Cat Swarm Optimization algorithm (ISCSO), Sand Cat Swarm Optimization algorithm (SCSO), Sparrow Search Algorithm (SSA) [32], Moth-Flame Optimization algorithm (MFO) [33], Snake Optimization algorithm (SO) [34], Grey Wolf Optimization algorithm (GWO), and Sailfish Optimization algorithm (SFO) [35]. The table includes the number of iterations, population size, and specific parameter configurations for each algorithm. The parameter settings are determined based on standard practices in metaheuristic optimization and feature selection, as well as preliminary experimental verification. Population size and maximum iterations are set to ensure both search performance and computational efficiency. Parameters of the comparison algorithms are adopted from their original papers to ensure a fair comparison.

### 4.3. Experiment 1: Global Optimization with CEC2017 Test Functions

The CEC 2017 test function set is a benchmark test function set released by the International Conference on Evolutionary Computation, used to evaluate the performance of evolutionary algorithms and intelligent optimization algorithms. The function set contains 29 test functions, where F1–F2 are unimodal functions, F3–F9 are simple multimodal functions, F10–F19 are hybrid functions, and F20–29 are composite functions.The details of the test function suite are presented in Table 2. Unimodal functions are used to test the local search capability of the algorithm; simple multimodal functions are used to inspect the algorithm’s ability to escape local optima and evaluate population diversity and global exploration performance; hybrid functions are used to examine the algorithm’s adaptability in complex hybrid search spaces; composite functions are used to evaluate the algorithm’s optimization capability in multi-level, multi-feature composite problems.

To comprehensively evaluate the effectiveness of the ISCSO algorithm, six commonly used heuristic algorithms are selected for comparison with ISCSO on CEC2017 test functions, including standard SCSO, MFO, SSA, SO, GWO, and SFO. All algorithms are evaluated under the same experimental conditions to maintain the integrity and consistency of the results. Each algorithm uses 30 search agents and performs 1000 iterations. To minimize the impact of random initialization on the results, each algorithm is independently executed 30 times, and performance is evaluated by average fitness value and standard deviation.

#### 4.3.1. Numerical and Statistical Analysis of Experiment 1

Table 3 shows the average fitness and standard deviation of ISCSO and other optimization algorithms on CEC2017 test functions. From the ranking of average fitness, ISCSO outperforms other optimization algorithms on 22 test functions. Specifically, ISCSO ranks first on F1-F7, F9-F14, F17, F18, F20-F24, F27, and F29; ranks second on F15, F16, F25, and F26; and ranks third on F8 F19 and F28. The results indicate that ISCSO possesses excellent local search capability and a strong ability to escape local optima, along with good adaptability in complex hybrid search spaces and effective optimization performance in multi-level, multi-feature composite problems. Overall, the performance of the ISCSO algorithm is better than the six compared algorithms.

In addition, in this test, ISCSO performs significantly better than SCSO. Specifically, ISCSO outperforms SCSO on 27 test functions, while SCSO outperforms ISCSO only on F8 and F19. It can be seen that the added strategies significantly improve the performance of the SCSO algorithm.

The Wilcoxon test [36] is widely used in statistical analysis to compare differences between two algorithms. Table 4 records the *p*-values of the Wilcoxon test between ISCSO and existing optimization algorithms. These *p*-values indicate whether there is a significant difference in performance between the two algorithms under various test functions. When the *p*-value is less than 0.05, it indicates that the difference between ISCSO and the compared algorithm in this test function is statistically significant. Conversely, if the *p*-value is 0.05 or higher, it indicates no significant performance difference between the two algorithms for the given test function, as shown below.

#### 4.3.2. Analysis of Experiment 1 Results

**Figure 3:**
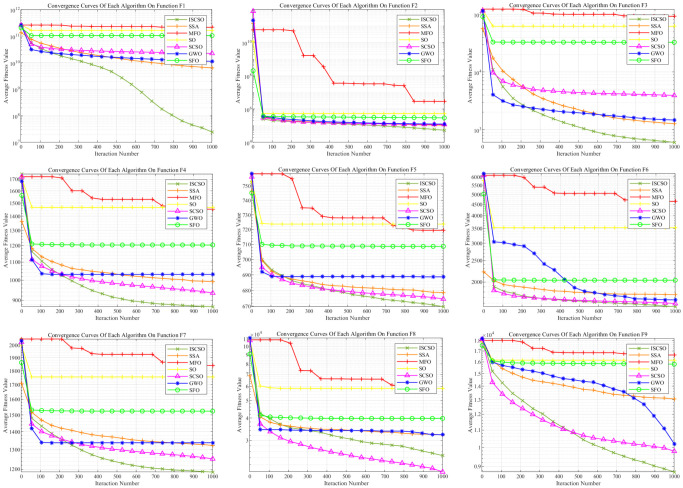

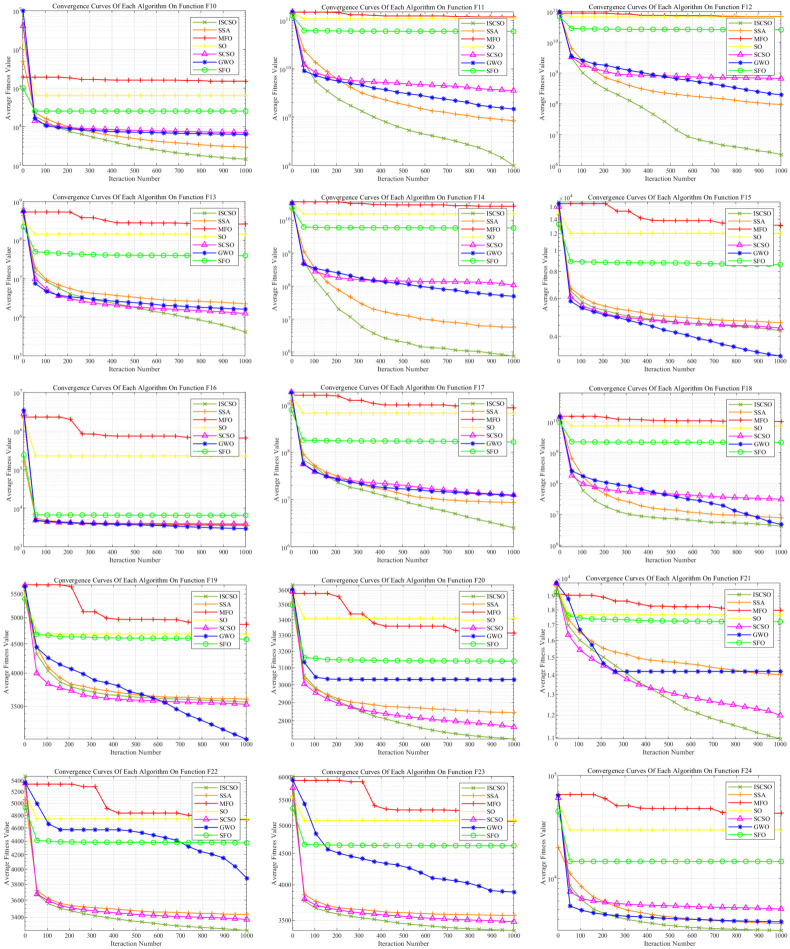

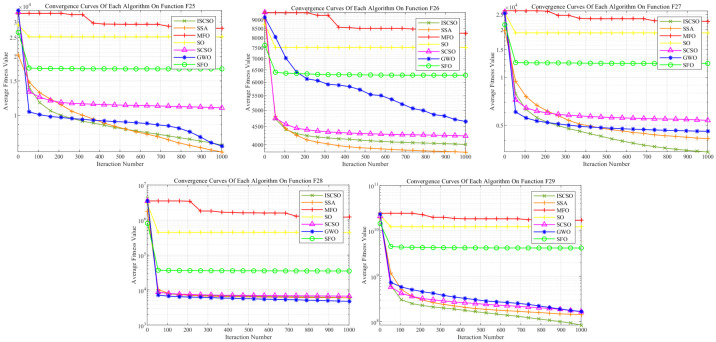
Convergence curves of CEC2017 test functions.

This section conducts an in-depth analysis of the performance of various optimization algorithms through convergence curves, evaluating their performance in solving CEC2017 test functions. By observing the convergence curves, we can intuitively understand the convergence speed and final convergence state of each algorithm during iteration. Figure 3 shows the convergence behavior of ISCSO compared with other optimization methods.

In functions F2, F5, F12, F14, F16, F18, F22, F23, F26, and F29, the convergence curve of ISCSO declines most rapidly during the early iterations, indicating high search efficiency, rapid exploration of the solution space, and a strong capacity to approach the optimal solution quickly. For the remaining test functions, although ISCSO is not the fastest-converging algorithm, it ranks second or third in convergence speed. The relatively slower convergence on some functions is attributed to their complex mathematical properties. Among them, F9 is a highly multimodal function with dense local optima; F15 and F16 are hybrid functions with interactive components and nonlinear variable coupling; F19 and F28 are high-dimensional composite functions formed by rotation, shifting, and composition of multiple basic functions, leading to highly irregular and non-convex search spaces. The fixed-intensity chaotic perturbation in ISCSO has limited adaptability to such extremely complex landscapes, resulting in slightly insufficient optimization performance on these individual functions. In most test functions (except F9, F15, F16, F19, and F28), the final stable fitness value of ISCSO is significantly lower than that of all other algorithms, indicating that ISCSO can obtain higher-precision solutions. In contrast, other algorithms tend to become trapped in local optima, and their convergence curves plateau without further decline as iterations increase. On the aforementioned functions where ISCSO does not reach the absolute best performance, it still obtains competitive results, mainly owing to the exponentially decaying nonlinear adaptive weight with Gaussian perturbation: a larger weight in the early stage strengthens global exploration, while a smaller weight in the later stage intensifies local exploitation (e.g., ISCSO converges rapidly to high-precision solutions in composite functions such as F12 and F14). Meanwhile, the Metropolis acceptance criterion accepts inferior solutions with a certain probability, and combined with the chaotic perturbation term, it significantly enhances the local optima escape ability of the algorithm in multimodal functions such as F9 and F18. Furthermore, ISCSO shows smaller fluctuations and smoother convergence curves with weaker random interference. The continuity of the golden sine mechanism ensures a controllable solution variation range during iterations, avoiding severe fitness oscillations caused by abrupt updates in traditional swarm intelligence algorithms. Compared with other algorithms (e.g., MFO with large fluctuations and SSA prone to premature stagnation), ISCSO exhibits stronger and more reliable stability.

Based on the above analysis, compared with other swarm intelligence optimization algorithms (SCSO, SSA, MFO, SO, GWO, SFO), ISCSO exhibits faster convergence (characterized by a rapid decrease), higher accuracy (yielding a lower final value), and better stability (manifested as small fluctuations and continuous optimization). Overall, ISCSO performs best on CEC2017 test functions compared to the six algorithms.

### 4.4. Experiment 2: Feature Selection

#### 4.4.1. Collection and Preprocessing of Feature Selection Datasets

**Figure 4:**
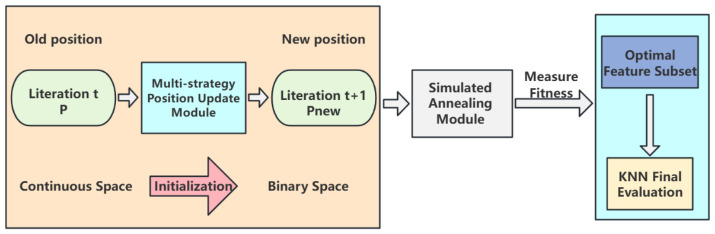
The feature selection process of ISCSO.

The feature selection experiment in this study uses 18 datasets from the well-known UCI Machine Learning Repository [37]. These datasets span a diverse range of research and application domains, including biology, medicine, physics, and social sciences, which enables sufficient verification of the effectiveness and generalization ability of feature selection approaches across various fields. Such a dataset selection strategy helps to comprehensively evaluate the performance, effectiveness, and universality of optimization-based feature selection algorithms when dealing with different types of data. Meanwhile, the number of samples in these datasets ranges from just over 100 to several thousand, covering both small-sample and large-sample scenarios. Datasets with varying scales allow researchers to systematically analyze the performance discrepancies of feature selection methods, as well as their computational efficiency and scalability when handling large-scale high-dimensional data. Furthermore, the selected datasets involve both binary classification and multi-classification tasks. These diverse task types can be used to rigorously test the adaptability of feature selection methods to different classification objectives and their feature extraction performance under varying levels of difficulty. The feature selection process of ISCSO is shown in Figure 4. The detailed descriptions and statistical information of each dataset are listed in Table 5.

(24) $$TF=\frac{1}{1+e^{-X_{i,d}}}$$

(25) $$X_{i,d}^{\mathrm{bin}}=\left\{\begin{array}{cc} 1, & \mathrm{if}\;TF\geq r\\ 0, & \mathrm{if}\;TF<r \end{array}\right.$$

Binarization is an important step in feature selection, used to convert the continuous position of the search agent (representing the probability of feature selection) into a binary decision (0/1 indicates whether the feature is selected) [38]. The mathematical principle is shown in Equations (24) and (25), where *r* represents a random number in the interval [0,1], and $X_{i,d}^{\mathrm{bin}}$ represents the binarized position, with 0 indicating that the feature is not selected and 1 indicating that it is selected. For datasets with missing values, numerical missing values are filled with the median, and categorical missing values are filled with the mode. The dataset is divided into a training set and a test set in an 8:2 ratio, and the KNN algorithm [39] (K = 5) is applied. All algorithms are evaluated under the same experimental conditions to ensure fairness and comparability. Each algorithm uses 30 search agents for 100 iterations. To reduce the impact of random initialization, each algorithm is independently executed 20 times. All datasets are divided into training and test sets with an 8:2 stratified split to preserve the class distribution. The KNN classifier (K = 5) is used for evaluation. To ensure reliability and reproducibility, each algorithm runs independently 20 times for feature selection tasks. Stratified sampling is adopted to avoid bias caused by imbalanced data distribution.

#### 4.4.2. Numerical and Statistical Analyses of Experiment 2

**Figure 5:**
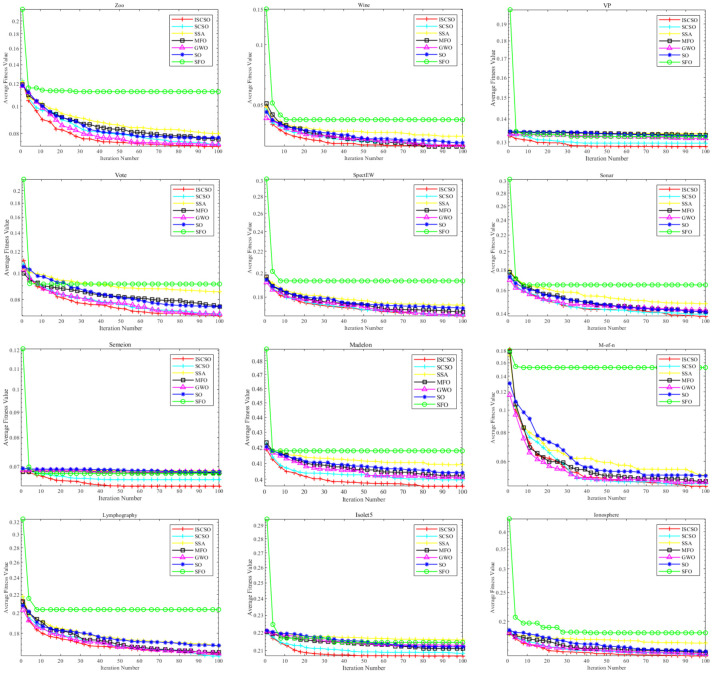

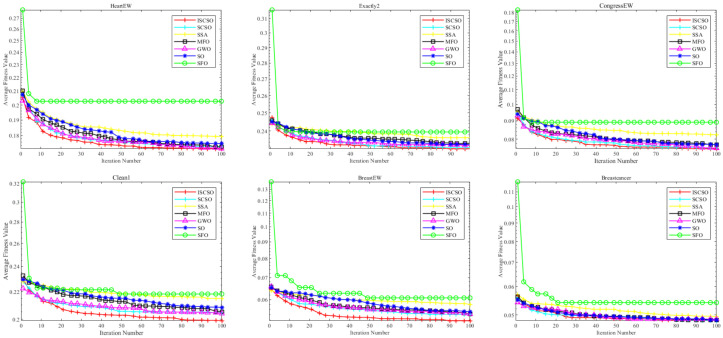
Convergence curves of the fitness function for feature selection.

This section evaluates the performance of ISCSO in feature selection using various indicators. Table 5, Table 6 and Table 7 show the comparison between ISCSO and other optimization algorithms on different datasets, and the convergence curves are shown in Figure 5. To highlight the optimal performance of the algorithm, the optimal values in the tables are shown in bold. To more clearly compare the overall performance, the last few rows summarize the overall ranking of each algorithm on 18 datasets.

Table 6 shows the average fitness of various optimization methods on each dataset. Among the 18 tested datasets, ISCSO achieves the optimal average fitness on 17 datasets, accounting for 94.44%, showing significant advantages. Only in the M-of-n dataset, the average fitness of ISCSO is slightly lower than that of the SO optimization algorithm. This can be attributed to the clear linear relationship among features in the M-of-n dataset, whereas the chaotic perturbation and golden sine strategy of ISCSO are more suitable for handling nonlinear problems, resulting in excessive searching in low-dimensional linear spaces. However, the difference between the two is small and has a limited impact on the overall results. In addition, compared with the original SCSO, ISCSO performs particularly well, outperforming SCSO in all datasets. Furthermore, the overall ranking shows that ISCSO is the highest-ranked and optimal-performing algorithm, while SFO is the lowest-ranked. Further referring to Convergence curves of the fitness function, which intuitively shows the average fitness of different optimization algorithms on 18 datasets, it can be clearly seen that ISCSO has lower average fitness values and better effects.

Table 7 provides detailed data on the number of features selected by each optimization method. The number of selected features is a key indicator for evaluating the effectiveness of the optimization method [40]. The fewer features selected, the stronger the algorithm’s ability to identify and eliminate redundant and irrelevant features is. This can effectively reduce the data dimension, avoid the “curse of dimensionality”, and improve computational efficiency.

The data in the table clearly show that ISCSO selects the fewest features on average among the 12 datasets. In the Sonar, Semeion, Madelon, Isolet5, Clean1, and SpectEW datasets, the number of selected features is lower than that of SFO, ranking second. This is because the computational cost of chaotic perturbation increases in high-dimensional scenarios, causing the algorithm to converge prematurely and fail to further eliminate redundant features. Although in terms of the number of selected features, the ISCSO algorithm is inferior to the SFO algorithm in some cases, in terms of standard deviation, ISCSO shows the lowest standard deviation among the 12 datasets, and its performance in other datasets is basically in the top three, indicating excellent stability and robustness. The reason why ISCSO cannot achieve optimal stability on the other six datasets is that its improved mechanism with strong exploration ability, high population diversity, and excellent premature convergence resistance is more adaptive to high-dimensional, complex, and multimodal problems, and it is prone to excessive oscillation and unstable search performance on low-dimensional, smooth, and low-noise datasets. In contrast, SFO shows the highest standard deviation in 13 datasets, indicating poor stability.

**Figure 6:**
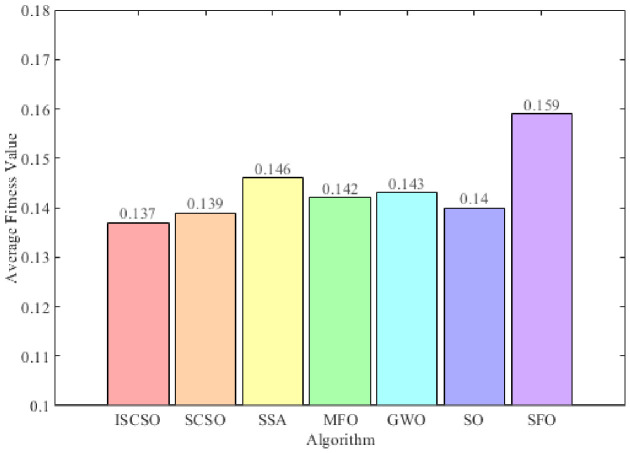
Average fitness of feature selection.

**Figure 7:**
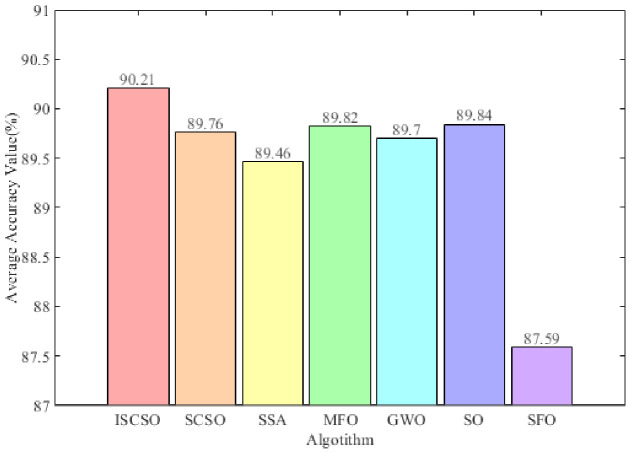
Average accuracy of feature selection.

In addition, to comprehensively validate the effectiveness and superiority of the proposed ISCSO method in feature selection applications, a comparative analysis was conducted on the average classification accuracy of feature subsets selected by different optimization algorithms, with the detailed results presented in Table 8. The statistical analysis demonstrates that the ISCSO algorithm attains the highest average classification accuracy across 16 out of 18 tested datasets, which strongly verifies that the multi-strategy fusion mechanism designed in this study yields a significant performance improvement. It should be noted that the classification accuracy of ISCSO on the M-of-n and Breastcancer datasets is slightly suboptimal compared with several competing algorithms. This phenomenon can be attributed to the fact that ISCSO excessively prioritizes global exploration capability and population diversity maintenance, which correspondingly weakens its local exploitation accuracy during the late iteration stage. Consequently, when dealing with low-dimensional datasets characterized by relatively simple feature distribution and weak nonlinear correlation, the algorithm tends to suffer from inadequate convergence accuracy. Meanwhile, the experimental results reveal that the SFO algorithm achieves the lowest average classification accuracy among all comparison methods on 16 datasets, reflecting its relatively poor feature selection capability. Therefore, despite the fact that some alternative algorithms select a smaller number of features on individual datasets, classification accuracy remains the primary and most critical evaluation criterion in practical feature selection tasks. In the process of eliminating redundant and irrelevant features, the negative influence on classification accuracy should be minimized to ensure the reliability of selected feature subsets. Overall, the proposed ISCSO algorithm achieves higher average classification accuracy than all the other six state-of-the-art optimization methods involved in the comparison. Furthermore, the average classification accuracy histogram of the 18 datasets is intuitively depicted in Figure 6. Average accuracy of feature selection is shown in Figure 7.

To further investigate the contribution of each improved module in the ISCSO algorithm and verify their mechanisms on datasets of varying complexity, ablation experiments were conducted on the high-dimensional complex dataset Isolet5 and the low-dimensional simple dataset Wine. Using the original SCSO algorithm as the baseline, we gradually introduced five comparative versions: SCSO, SCSO+C (Chaotic Map), SCSO+C+G (Golden Sine Algorithm), SCSO+C+G+W (Adaptive Weigh), and the complete ISCSO. The average fitness and standard deviation (std) of each version on the two datasets are presented in the corresponding Table 9. The comprehensive ablation experiment results on the two datasets demonstrate that the improved modules of ISCSO reveal distinct application boundaries: in high-dimensional complex scenarios (e.g., Isolet5), each module significantly enhances the algorithm’s performance through synergy. Therefore, the complete ISCSO algorithm exhibits the optimal performance in terms of fitness and standard deviation on high-dimensional datasets. In low-dimensional simple scenarios (e.g., Wine), however, the strong exploration characteristics of some modules introduce search noise, which renders the full ISCSO algorithm inferior to the version integrated with only partial modules.

## 5. Conclusions

This paper proposes an improved Sand Cat Swarm Optimization algorithm (ISCSO) for global optimization and feature selection by integrating multiple complementary strategies within a unified optimization framework. The proposed method effectively enhances population diversity, achieves a balance between exploration and exploitation, and strengthens the capacity to escape local optima. Extensive experimental evaluations on the CEC2017 benchmark suite and UCI datasets demonstrate that ISCSO consistently outperforms existing metaheuristic algorithms in terms of convergence speed, solution accuracy, and robustness. The statistical analysis further confirms the significance and stability of the proposed approach across different problem types, including unimodal, multimodal, and composite functions, as well as high-dimensional feature selection tasks.

## Figures and Tables

**Table 1:** Parameter Settings.

Algorithm	Parameter
Overall	Global optimization: Population size = 30, Maximum iterations = 1000, Run count = 30
	Feature selection parameters: Population size = 30, Maximum iterations = 100, Run count = 20
ISCSO	w_max = 0.9, w_min = 0.4, k = 2, sigma = 0.1, S = 2, goldenRatio = (sqrt(5) − 1)/2, p = [1:360], alpha = 0.95
SCSO	S = 2, p = [1:360]
SSA	-
MFO	b = 1
SO	vec_flag = [−1,1], Threshold = 0.25, Threshold2 = 0.6, C1 = 0.5, C2 = 0.05, C3 = 2
GWO	a = 2, C1/C2/C3 = [0,2], A1/A2/A3 = [−a,a]
SFO	PD = 2/3, ks = 0.5/Maxit, NF = 2*npop

**Table 2:** Details of CEC 2017 test functions.

Func	Type	Theoretical Global Optimum
F1	Shifted and Rotated Bent Cigar Function	100
F2	Shifted and Rotated Zakharov Function	200
F3	Shifted and Rotated Rosenbrock Function	300
F4	Shifted and Rotated Rastrigin Function	400
F5	Shifted and Rotated Expanded Scaffer F6 Function	500
F6	ShiftedandRotatedLunacek Bi Rastrigin Function	600
F7	Shifted and Rotated NonContinuous Rastrigin Function	700
F8	Shifted and Rotated Levy Function	800
F9	Shifted and Rotated Schwefel Function	900
F10	Hybrid Function 1 (N = 3)	1000
F11	Hybrid Function 2 (N = 3)	1100
F12	Hybrid Function 3 (N = 3)	1200
F13	Hybrid Function 4 (N = 4)	1300
F14	Hybrid Function 5 (N = 4)	1400
F15	Hybrid Function 6 (N = 4)	1500
F16	Hybrid Function 6 (N = 5)	1600
F17	Hybrid Function 6 (N = 5)	1700
F18	Hybrid Function 6 (N = 5)	1800
F19	Hybrid Function 6 (N = 6)	1900
F20	Composition Function 1 (N = 3)	2000
F21	Composition Function 2 (N = 3)	2100
F22	Composition Function 3 (N = 4)	2200
F23	Composition Function 4 (N = 4)	2300
F24	Composition Function 5 (N = 5)	2400
F25	Composition Function 6 (N = 5)	2500
F26	Composition Function 7 (N = 6)	2600
F27	Composition Function 8 (N = 6)	2700
F28	Composition Function 9 (N = 3)	2800
F29	Composition Function 10 (N = 3)	2900

**Table 3:** Results of CEC2017 test functions.

Func		ISCSO	SSA	MFO	SO	SCSO	GWO	SFO
F1	avg	$2.46\times 10^{7}$	$6.39\times 10^{9}$	$2.15\times 10^{11}$	$1.65\times 10^{11}$	$2.24\times 10^{10}$	$1.13\times 10^{10}$	$1.04\times 10^{11}$
F1	std	$3.61\times 10^{6}$	$1.33\times 10^{9}$	$2.39\times 10^{10}$	$2.44\times 10^{10}$	$7.25\times 10^{9}$	$3.47\times 10^{9}$	$7.63\times 10^{9}$
F2	avg	$5.4\times 10^{4}$	$1.08\times 10^{5}$	$2.91\times 10^{6}$	$5.45\times 10^{5}$	$1.18\times 10^{5}$	$1.26\times 10^{5}$	$2.96\times 10^{5}$
F2	std	$9.71\times 10^{3}$	$1.28\times 10^{4}$	$9.22\times 10^{6}$	$1.57\times 10^{5}$	$1.91\times 10^{4}$	$2.41\times 10^{4}$	$6.11\times 10^{4}$
F3	avg	602.33	1269.27	$9.25\times 10^{4}$	$6.2\times 10^{4}$	3923.71	1469.1	$3.27\times 10^{4}$
F3	std	50.48	284.51	$1.97\times 10^{4}$	$1.95\times 10^{4}$	1335.53	553.67	3972
F4	avg	869.97	992.24	1452.56	1466.51	934.8	1031.05	1203.48
F4	std	35.86	40.37	96.75	94.61	35.36	66.9	45.95
F5	avg	669.78	678.71	719.46	723.66	674.62	688.78	708.71
F5	std	5.78	8.26	13.75	9.5	8.33	7.27	9.36
F6	avg	1544.18	1751.36	4645.02	3531.61	1595.84	1657.59	2036.57
F6	std	115.64	155	893.48	555.96	129.8	122.46	75.54
F7	avg	1185.77	1323.57	1840.46	1754.79	1251.87	1337.8	1524.41
F7	std	44.87	40.13	121.34	99.14	40.05	71.49	39.83
F8	avg	$2.53\times 10^{4}$	$3.32\times 10^{4}$	$5.66\times 10^{4}$	$5.88\times 10^{4}$	$1.95\times 10^{4}$	$1.05\times 10^{4}$	$4.06\times 10^{4}$
F8	std	3683.06	3203.23	$1.03\times 10^{4}$	9685.48	3762.27	4582.95	5995.15
F9	avg	8732.45	$1.3\times 10^{4}$	$1.66\times 10^{4}$	$1.61\times 10^{4}$	9789.58	$1.01\times 10^{4}$	$1.58\times 10^{4}$
F9	std	1031.83	1057.52	799.05	683.43	1008.28	1709.98	975.36
F10	avg	1454.15	2977.61	$1.5\times 10^{5}$	$6.45\times 10^{4}$	7139.08	6372.47	$2.53\times 10^{4}$
F10	std	66.06	461.26	$7.55\times 10^{4}$	$3.77\times 10^{4}$	2547.77	2406.05	3003
F11	avg	$7.91\times 10^{7}$	$8.42\times 10^{7}$	$1.18\times 10^{11}$	$1.08\times 10^{11}$	$3.53\times 10^{9}$	$1.47\times 10^{9}$	$5.97\times 10^{10}$
F11	std	$3.27\times 10^{7}$	$3.48\times 10^{8}$	$2.48\times 10^{10}$	$2.44\times 10^{10}$	$4\times 10^{9}$	$1.69\times 10^{9}$	$1.45\times 10^{10}$
F12	avg	$2.27\times 10^{6}$	$9.57\times 10^{7}$	$6.84\times 10^{10}$	$6.63\times 10^{10}$	$6.59\times 10^{8}$	$1.97\times 10^{8}$	$2.54\times 10^{10}$
F12	std	$4.69\times 10^{5}$	$5.16\times 10^{7}$	$2.38\times 10^{10}$	$1.83\times 10^{10}$	$1.14\times 10^{9}$	$1.5\times 10^{8}$	$8.93\times 10^{9}$
F13	avg	$4.14\times 10^{5}$	$2.24\times 10^{6}$	$2.6\times 10^{8}$	$1.42\times 10^{8}$	$1.26\times 10^{6}$	$1.62\times 10^{6}$	$3.96\times 10^{7}$
F13	std	$2.72\times 10^{5}$	$1.48\times 10^{6}$	$2.21\times 10^{8}$	$1.24\times 10^{8}$	$1.29\times 10^{6}$	$1.54\times 10^{6}$	$3.93\times 10^{7}$
F14	avg	$7.64\times 10^{5}$	$5.65\times 10^{6}$	$2.65\times 10^{10}$	$1.55\times 10^{10}$	$1.07\times 10^{8}$	$4.94\times 10^{7}$	$5.82\times 10^{9}$
F14	std	$1.82\times 10^{5}$	$2.85\times 10^{6}$	$9.25\times 10^{9}$	$7.11\times 10^{9}$	$2.63\times 10^{8}$	$1.36\times 10^{8}$	$3.54\times 10^{9}$
F15	avg	4246.35	4627.57	$1.31\times 10^{4}$	$1.2\times 10^{4}$	4366.87	3242.02	8626.58
F15	std	499.22	485.19	2560.73	2113.74	612.64	378.63	1679.45
F16	avg	3559.92	3772.51	$6.55\times 10^{5}$	$2.26\times 10^{5}$	3910.41	2969.91	6552.46
F16	std	459.59	319.07	$6.89\times 10^{5}$	$4.23\times 10^{5}$	299.61	307.01	2352.94
F17	avg	$2.53\times 10^{6}$	$8.67\times 10^{6}$	$8.73\times 10^{8}$	$6.72\times 10^{8}$	$1.25\times 10^{7}$	$1.23\times 10^{7}$	$1.65\times 10^{8}$
F17	std	$1.49\times 10^{6}$	$7.89\times 10^{6}$	$5.18\times 10^{8}$	$6.05\times 10^{8}$	$1.41\times 10^{7}$	$1.35\times 10^{7}$	$1.12\times 10^{8}$
F18	avg	$4.11\times 10^{6}$	$7.78\times 10^{6}$	$1.02\times 10^{10}$	$7.31\times 10^{9}$	$3.06\times 10^{7}$	$4.67\times 10^{6}$	$2.14\times 10^{9}$
F18	std	$1.97\times 10^{6}$	$4.5\times 10^{6}$	$2.9\times 10^{9}$	$3.19\times 10^{9}$	$6.34\times 10^{7}$	$7.69\times 10^{6}$	$1.42\times 10^{9}$
F19	avg	3560.93	3605.4	4867.44	4682.06	3528.33	3068.26	4583.2
F19	std	334.42	342.78	289.67	276.89	310.56	298.45	267.9
F20	avg	5677.21	5890.12	8774.43	8134.55	5887.63	5210.1	7654.3
F20	std	454.79	477.9	652.33	597.67	489.01	422.1	577.82
F21	avg	6559.94	6901.23	9796.54	9355.67	7099.76	6621.09	8864.43
F21	std	566.79	589.07	766.43	709.87	591.12	543.11	679.9
F22	avg	7890.55	8012.33	$1.09\times 10^{4}$	$1.04\times 10^{4}$	8109.88	7430.1	9836.74
F22	std	677.9	690.22	876.14	810.17	691.98	654.19	789.01
F23	avg	8901.56	9123.43	$1.2\times 10^{4}$	$1.15\times 10^{4}$	9210.98	8543.21	$1.09\times 10^{4}$
F23	std	789.16	801.23	972.98	921.09	792.35	764.1	890.12
F24	avg	9012.67	9231.56	$1.32\times 10^{4}$	$1.26\times 10^{4}$	$1.03\times 10^{4}$	9673.32	$1.2\times 10^{4}$
F24	std	890.12	912.24	1098.72	1032.1	893.44	876.5	1001.22
F25	avg	$1.01\times 10^{4}$	$1.03\times 10^{4}$	$1.43\times 10^{4}$	$1.37\times 10^{4}$	$1.14\times 10^{4}$	$1.07\times 10^{4}$	$1.32\times 10^{4}$
F25	std	901.23	927.45	1209.81	1148.21	904.5	988.65	1112.37
F26	avg	$1.12\times 10^{4}$	$1.14\times 10^{4}$	$1.53\times 10^{4}$	$1.48\times 10^{4}$	$1.25\times 10^{4}$	$1.18\times 10^{4}$	$1.43\times 10^{4}$
F26	std	1012.35	1030.56	1320.95	1254.39	1015.6	1098.73	1223.58
F27	avg	$1.24\times 10^{4}$	$1.25\times 10^{4}$	$1.65\times 10^{4}$	$1.59\times 10^{4}$	$1.36\times 10^{4}$	$1.29\times 10^{4}$	$1.54\times 10^{4}$
F27	std	1123.47	1145.6	1432.07	1363.43	1126.01	1209.85	1334.58
F28	avg	$1.34\times 10^{4}$	$1.36\times 10^{4}$	$1.76\times 10^{4}$	$1.7\times 10^{4}$	$1.47\times 10^{4}$	$1.4\times 10^{4}$	$1.64\times 10^{4}$
F28	std	1235.13	1256.78	1543.2	1476.54	1237.89	1320.98	1445.67
F29	avg	$1.45\times 10^{4}$	$1.47\times 10^{4}$	$1.87\times 10^{4}$	$1.81\times 10^{4}$	$1.58\times 10^{4}$	$1.52\times 10^{4}$	$1.76\times 10^{4}$
F29	std	1346.68	1367.89	1654.32	1587.65	1348.9	1432.09	1556.78

Underlined values represent the optimal values for each function.

**Table 4:** Wilcoxon test results.

Function	SSA	MFO	SO	SCSO	GWO	SFO
F1	3.02 × 10^−11^	3.02 × 10^−11^	3.02 × 10^−11^	3.02 × 10^−11^	3.02 × 10^−11^	3.02 × 10^−11^
F2	3.02 × 10^−11^	3.02 × 10^−11^	3.02 × 10^−11^	6.70 × 10^−11^	3.69 × 10^−11^	3.02 × 10^−11^
F3	3.02 × 10^−11^	3.02 × 10^−11^	3.02 × 10^−11^	3.02 × 10^−11^	3.02 × 10^−11^	3.02 × 10^−11^
F4	3.02 × 10^−11^	3.02 × 10^−11^	3.02 × 10^−11^	2.15 × 10^−10^	3.82 × 10^−09^	3.02 × 10^−11^
F5	2.20 × 10^−07^	3.02 × 10^−11^	3.02 × 10^−11^	1.68 × 10^−04^	3.02 × 10^−11^	3.34 × 10^−11^
F6	1.25 × 10^−04^	3.02 × 10^−11^	3.02 × 10^−11^	9.07 × 10^−03^	3.02 × 10^−11^	3.34 × 10^−11^
F7	5.49 × 10^−11^	3.02 × 10^−11^	3.02 × 10^−11^	1.09 × 10^−05^	6.72 × 10^−10^	3.02 × 10^−11^
F8	2.23 × 10^−09^	3.02 × 10^−11^	3.02 × 10^−11^	1.11 × 10^−06^	8.99 × 10^−11^	7.39 × 10^−11^
F9	3.69 × 10^−11^	3.02 × 10^−11^	3.02 × 10^−11^	4.98 × 10^−04^	2.28 × 10^−05^	3.02 × 10^−11^
F10	3.02 × 10^−11^	3.02 × 10^−11^	3.02 × 10^−11^	3.02 × 10^−11^	3.02 × 10^−11^	3.02 × 10^−11^
F11	3.02 × 10^−11^	3.02 × 10^−11^	3.02 × 10^−11^	4.98 × 10^−11^	5.07 × 10^−10^	3.02 × 10^−11^
F12	3.02 × 10^−11^	3.02 × 10^−11^	3.02 × 10^−11^	6.07 × 10^−11^	3.02 × 10^−11^	3.02 × 10^−11^
F13	3.20 × 10^−09^	3.02 × 10^−11^	3.02 × 10^−11^	1.89 × 10^−04^	8.15 × 10^−05^	3.02 × 10^−11^
F14	3.02 × 10^−11^	3.02 × 10^−11^	3.02 × 10^−11^	2.71 × 10^−02^	1.86 × 10^−01^	3.02 × 10^−11^
F15	5.32 × 10^−03^	3.02 × 10^−11^	3.02 × 10^−11^	3.87 × 10^−01^	2.23 × 10^−09^	3.02 × 10^−11^
F16	7.24 × 10^−02^	3.02 × 10^−11^	3.02 × 10^−11^	2.27 × 10^−03^	1.73 × 10^−06^	1.78 × 10^−10^
F17	2.00 × 10^−05^	3.02 × 10^−11^	3.02 × 10^−11^	1.89 × 10^−04^	4.12 × 10^−06^	3.02 × 10^−11^
F18	4.22 × 10^−04^	3.02 × 10^−11^	3.02 × 10^−11^	2.01 × 10^−01^	2.61 × 10^−02^	3.02 × 10^−11^
F19	5.79 × 10^−01^	3.02 × 10^−11^	3.69 × 10^−11^	5.49 × 10^−01^	4.42 × 10^−06^	2.61 × 10^−10^
F20	5.97 × 10^−09^	3.02 × 10^−11^	3.02 × 10^−11^	9.07 × 10^−03^	1.86 × 10^−09^	3.02 × 10^−11^
F21	3.02 × 10^−11^	3.02 × 10^−11^	3.02 × 10^−11^	2.32 × 10^−06^	8.12 × 10^−04^	3.02 × 10^−11^
F22	3.37 × 10^−05^	3.02 × 10^−11^	3.02 × 10^−11^	1.91 × 10^−02^	2.61 × 10^−10^	3.02 × 10^−11^
F23	4.12 × 10^−06^	3.02 × 10^−11^	3.02 × 10^−11^	6.97 × 10^−03^	5.09 × 10^−08^	3.02 × 10^−11^
F24	3.02 × 10^−11^	3.02 × 10^−11^	3.02 × 10^−11^	3.02 × 10^−11^	3.02 × 10^−11^	3.02 × 10^−11^
F25	5.89 × 10^−01^	3.02 × 10^−11^	3.02 × 10^−11^	8.88 × 10^−06^	4.38 × 10^−01^	3.02 × 10^−11^
F26	7.62 × 10^−03^	3.02 × 10^−11^	3.02 × 10^−11^	1.52 × 10^−03^	1.20 × 10^−08^	3.34 × 10^−11^
F27	3.02 × 10^−11^	3.02 × 10^−11^	3.02 × 10^−11^	3.02 × 10^−11^	3.02 × 10^−11^	3.02 × 10^−11^
F28	9.35 × 10^−01^	3.02 × 10^−11^	3.02 × 10^−11^	5.26 × 10^−04^	9.76 × 10^−10^	3.02 × 10^−11^
F29	6.52 × 10^−09^	3.02 × 10^−11^	3.02 × 10^−11^	2.77 × 10^−05^	2.39 × 10^−08^	3.02 × 10^−11^

**Table 5:** Dataset details.

Dataset	Feature Count	Sample Count	Classes
Zoo	16	101	7
Wine	13	178	3
VP	128	669	3
Vote	16	300	2
SpectEW	22	267	2
Sonar	60	208	2
Semeion	256	1593	10
M-of-n	13	1000	2
Madelon	500	2600	2
Lymphography	18	148	4
Isolet5	617	7797	26
Ionosphere	34	351	2
juHeartEW	13	270	2
Exactly2	13	1000	2
CongressEW	16	434	2
Clean1	166	476	2
BreastEW	30	568	2
Breastcancer	32	569	2

**Table 6:** Average fitness of feature selection.

Dataset	ISCSO-KNN	SCSO-KNN	SSA-KNN	MFO-KNN	GWO-KNN	SO-KNN	SFO-KNN
Zoo	**0.0784**	0.0828	0.0886	0.0856	0.0846	0.0811	0.1142
Wine	**0.0328**	0.034	0.0371	0.0345	0.0353	0.0343	0.04409
VP	**0.1288**	0.13	0.1337	0.1335	0.1335	0.1323	0.1334
Vote	**0.0661**	0.0665	0.075	0.0714	0.0693	0.0663	0.0844
SpectEW	**0.1719**	0.1723	0.1784	0.1748	0.1765	0.1732	0.1946
Sonar	**0.1457**	0.1459	0.1553	0.1499	0.1494	0.148	0.167
Semeion	**0.0647**	0.0663	0.0687	0.0682	0.0687	0.0684	0.0683
M-of-n	0.0577	0.058	0.0646	0.0592	0.0634	**0.0554**	0.15293
Madelon	**0.3994**	0.4029	0.4121	0.4072	0.4087	0.4054	0.4187
Lymphography	**0.1698**	0.1718	0.1783	0.1737	0.1772	0.1713	0.2054
Isolet5	**0.2085**	0.2105	0.2171	0.2138	0.214	0.2151	0.2164
Ionosphere	**0.1579**	0.1607	0.1731	0.164	0.1675	0.1612	0.1895
HeartEW	**0.1762**	0.1786	0.1852	0.1801	0.1822	0.1784	0.2037
Exactly2	**0.2328**	0.2333	0.239	0.2368	0.2361	0.2345	0.2405
CongressEW	**0.0779**	0.0787	0.0852	0.0811	0.0818	0.08	0.0905
Clean1	**0.2045**	0.2082	0.2192	0.2136	0.2154	0.2089	0.2214
BreastEW	**0.054**	0.0563	0.06	0.0575	0.0588	0.0571	0.0635
Breastcancer	**0.0495**	0.0496	0.0516	0.0503	0.05	0.0499	0.0557

Bold values denote the optimal results on the corresponding dataset.

**Table 7:** Average number of features selected.

Dataset		ISCSO	SCSO	SSA	MFO	GWO	SO	SFO
Zoo	Mean	**6.7333**	6.9	8	7.4	7.5667	6.9333	8.4
Zoo	Std	0.6617	0.7849	0.9097	**0.5632**	0.7279	0.7849	1.4527
Wine	Mean	4.0667	4.1	4.5333	**4.0399**	4.2	4.1	5.3333
Wine	Std	**0.1537**	0.3051	0.5713	0.1825	0.4068	0.3051	1.0283
VP	Mean	**55.3667**	55.9333	57.1333	59.4	60.9667	59.3333	55.8667
VP	Std	**4.4604**	6.3838	6.1236	6.0263	6.5783	6.3535	7.0109
Vote	Mean	**4**	**4**	4.5333	**4**	4.2667	4.0333	5.2667
Vote	Std	**0**	**0**	0.50741	**0**	0.4498	0.1826	1.0148
SpectEW	Mean	9.9	10.5	10.6667	10.8333	10.9667	10.4	**9.6667**
SpectEW	Std	1.4797	1.5613	1.4223	**1.3412**	1.3514	1.61	2.0398
Sonar	Mean	30.2333	31.7667	34.8667	34.1333	34.1333	32.8667	**27.6333**
Sonar	Std	**3.2236**	4.6659	3.8303	3.9211	4.7903	4.1583	3.8817
Semeion	Mean	123.567	128.5	127.367	127.933	131.233	131.7	**120.733**
Semeion	Std	**7.8198**	9.3246	8.4628	11.8523	14.431	12.41	14.7108
M-of-n	Mean	**6.1**	6.25	6.8	6.4	6.75	6.35	10.15
M-of-n	Std	**0.3077**	0.4442	0.4103	0.5026	0.4442	0.4893	1.5313
Madelon	Mean	253.2	287.2	309.15	310.2	304	316.95	**240**
Madelon	Std	31.0341	31.279	31.4764	18.6169	19.6147	**12.3436**	32.1149
Lymphography	Mean	**9.2333**	9.5667	9.9	9.6	9.5333	10.0333	9.2667
Lymphography	Std	1.4064	1.5447	1.6887	1.4527	**1.2242**	1.2994	1.4125
Isolet5	Mean	334.43	344.9	360.567	366.433	379.467	364.433	**306.433**
Isolet5	Std	29.367	30.142	45.7	31.144	29.885	29.683	**11.938**
Ionosphere	Mean	**12.5667**	12.8667	14.8	13.4333	14.0667	14.0333	13.3333
Ionosphere	Std	**1.7143**	2.08	2.4269	1.9241	1.7207	1.79	2.3835
HeartEW	Mean	**4.9667**	5.1667	5.6	5.6	5.2333	5.3667	5.4
HeartEW	Std	**1.2058**	1.4015	1.3287	1.3287	1.3565	1.3256	1.5919
Exactly2	Mean	**1.1**	1.1667	1.8667	1.4667	1.4	1.3333	2.2667
Exactly2	Std	**0.3051**	0.379	0.6288	0.5074	0.4983	0.4795	0.6915
CongressEW	Mean	**3.0667**	3.9333	4.8	4.2	4.2	4	4.9667
CongressEW	Std	**0.7396**	1.0482	1.0306	0.9613	0.8052	0.9097	1.4259
Clean1	Mean	91.9	92.4333	96.8333	100.2667	96.9	97.9333	**78.5**
Clean1	Std	9.1664	9.5725	11.1109	**6.0511**	7.9366	9.6416	13.528
BreastEW	Mean	**10.1667**	11.4	11.6333	11.1333	10.8667	11.1333	10.5333
BreastEW	Std	1.3025	1.4499	2.3265	1.5477	1.5698	**1.1665**	2.08
Breastcancer	Mean	**2**	2.0667	2.4667	2.1667	2.1667	2.2667	2.7667
Breastcancer	Std	**0.2537**	0.3051	0.5074	0.379	0.379	0.4497	0.504

Bold values denote the optimal results on the corresponding dataset.

**Table 8:** Average accuracy of feature selection.

Dataset	ISCSO-KNN	SCSO-KNN	SSA-KNN	MFO-KNN	GWO-KNN	SO-KNN	SFO-KNN
Zoo	**0.9667**	**0.9667**	**0.9667**	**0.9667**	**0.9667**	**0.9667**	0.9333
Wine	**1**	**1**	**1**	**1**	**1**	**1**	0.9987
VP	**0.9087**	0.9047	0.9012	0.904	0.9057	0.9053	0.9015
Vote	**1**	**1**	**1**	**1**	**1**	**1**	0.9969
SpectEW	**0.8712**	0.8654	0.8608	0.8675	0.865	0.8675	0.8342
Sonar	**0.9095**	0.9038	0.9	0.9059	0.9065	0.9027	0.8677
Semeion	**0.9821**	0.9807	0.9771	0.9781	0.9792	0.9794	0.9753
M-of-n	0.9998	0.9998	0.9998	**1**	0.9998	**1**	0.9189
Madelon	**0.6285**	0.6198	0.6143	0.6222	0.6186	0.6247	0.5793
Lymphography	**0.8858**	0.8811	0.8742	0.878	0.8712	0.8796	0.8311
Isolet5	**0.8371**	0.8304	0.8251	0.8315	0.8318	0.83	0.8169
Ionosphere	**0.8827**	0.8708	0.8606	0.8695	0.8702	0.8711	0.8403
HeartEW	0.8541	0.8498	0.8486	**0.8556**	0.8502	0.8547	0.821
Exactly2	**0.7533**	**0.7533**	**0.7533**	**0.7533**	**0.7533**	**0.7533**	**0.7533**
CongressEW	**0.9546**	0.9433	0.9418	0.9433	0.9433	0.9439	0.9354
Clean1	**0.8448**	0.8347	0.8261	0.8392	0.8333	0.839	0.81
BreastEW	**0.9871**	0.9822	0.9786	0.981	0.979	0.9809	0.9716
Breastcancer	0.9723	0.9719	**0.9754**	0.9728	0.9728	0.9738	0.974

Bold values denote the optimal results on the corresponding dataset.

**Table 9:** Result of ablation experiments.

Dataset	Algorithm	Fitness	Std
Wine	SCSO	0.04115	0.0054
Wine	SCSO+C	0.041	0.00683
Wine	SCSO+C+G	0.04159	0.00579
Wine	SCSO+C+G+W	**0.041**	**0.0051**
Wine	ISCSO	0.04	0.05038
Isolet5	SCSO	0.21143	0.00288
Isolet5	SCSO+C	0.21112	0.00284
Isolet5	SCSO+C+G	0.21056	0.00281
Isolet5	SCSO+C+G+W	0.20811	0.00211
Isolet5	ISCSO	**0.20596**	**0.0021**

Bold values denote the optimal results on the corresponding dataset.

## Data Availability

The raw data supporting the conclusions of this article will be made available by the authors on request.

## References

[1] Xue B., Zhang M., Browne W.N. (2013). Particle Swarm Optimization for Feature Selection in Classification: A Multi-Objective Approach. IEEE Trans. Cybern..

[2] Van Hulse J., Khoshgoftaar T.M., Napolitano A. A comparative evaluation of feature ranking methods for high dimensional bioinformatics data. Proceedings of the 2011 IEEE International Conference on Information Reuse & Integration.

[3] Kumar A., Yadav S.P., Kumar A. (2025). An improved feature extraction algorithm for robust Swin Transformer model in high-dimensional medical image analysis. Comput. Biol. Med..

[4] Urbanowicz R.J., Olson R.S., Schmitt P.A., Moore J.H. (2017). Benchmarking Relief-Based Feature Selection Methods for Bioinformatics Data Mining. J. Biomed. Inform..

[5] Yang M., Lim M.K., Qu Y., Li Z. (2023). Deep neural networks with L1 and L2 regularization for high dimensional corporate credit risk prediction. Expert Syst. Appl..

[6] Jeffery I.B., Higgins D.G., Culhane A.C. (2006). Comparison and evaluation of methods for generating differentially expressed gene lists from microarray data. BMC Bioinform..

[7] Bach F. (2017). Breaking the curse of dimensionality with convex neural networks. J. Mach. Learn. Res..

[8] Verleysen M., François D. (2005). The Curse of Dimensionality in Data Mining and Time Series Prediction. Computational Intelligence and Bioinspired Systems.

[9] Liu C., Wang W., Zhao Q., Shen X. (2017). A new feature selection method based on a validity index of feature subset. Pattern Recognit. Lett..

[10] Golub T.R., Slonim D.K., Tamayo P., Huard C., Gaasenbeek M., Mesirov J.P., Coller H., Loh M.L., Downing J.R., Caligiuri M.A. (1999). Molecular classification of cancer: Class discovery and class prediction by gene expression monitoring. Science.

[11] Kohavi R., John G.H. (1997). Wrappers for feature subset selection. Artif. Intell..

[12] Chu W., Gao X., Sorooshian S. (2011). A new evolutionary search strategy for global optimization of high-dimensional problems. Inf. Sci..

[13] Esmin A.A., Coelho R.A., Matwin S. (2015). A review on particle swarm optimization algorithm and its variants to clustering high-dimensional data. Artif. Intell. Rev..

[14] Lambora A., Gupta K., Chopra K. Genetic Algorithm- A Literature Review. Proceedings of the 2019 International Conference on Machine Learning, Big Data, Cloud and Parallel Computing.

[15] Kennedy J., Eberhart R. (1995). Particle swarm optimization. Proceedings of the ICNN’95 International Conference on Neural Networks, Perth, WA, Australia, 27 November–1 December 1995.

[16] Mirjalili S., Mirjalili S.M., Lewis A. (2014). Grey wolf optimizer. Adv. Eng. Softw..

[17] Seyyedabbasi A., Kiani F. (2023). Sand Cat swarm optimization: A nature-inspired algorithm to solve global optimization problems. Eng. Comput..

[18] Wu D., Rao H., Wen C., Jia H., Abualigah L. (2022). Modified sand cat swarm optimization algorithm for solving constrained engineering optimization problems. Mathematics.

[19] Kumar Y., Singh P.K. (2018). Improved cat swarm optimization algorithm for solving global optimization problems and its application to clustering. Appl. Intell..

[20] Jia H., Zhang J., Rao H., Wen C., Li C. (2024). Improved sandcat swarm optimization algorithm for solving global optimum problems. Artif. Intell. Rev..

[21] Kumar A. (2008). Computer-vision-based fabric defect detection: A survey. IEEE Trans. Ind. Electron..

[22] Canny J. (1986). A computational approach to edge detection. IEEE Trans. Pattern Anal. Mach. Intell..

[23] Hénon M. (1976). A two-dimensional mapping with a strange attractor. Commun. Math. Phys..

[24] Tanyildizi E., Demir G. (2017). Golden sine algorithm: A novel math-inspired algorithm. Adv. Electr. Comput. Eng..

[25] Li X., Yin M. (2014). Parameter estimation for chaotic systems by hybrid differential evolution algorithm and artificial bee colony algorithm. Nonlinear Dyn..

[26] Wang Z., Li Y., Wu L., Guo Q. (2024). A nonlinear adaptive weight-based mutated whale optimization algorithm and its application for solving engineering problems. IEEE Access.

[27] Kirkpatrick S., Gelatt C.D., Vecchi M.P. (1983). Optimization by simulated annealing. Science.

[28] Aarts E.H.L. (1987). Simulated Annealing: Theory and Applications.

[29] Wu G., Mallipeddi R., Suganthan P.N. (2017). Problem Definitions and Evaluation Criteria for the CEC 2017 Competition on Constrained Real-Parameter Optimization.

[30] Kwon O., Sim J.M. (2013). Effects of data set features on the performances of classification algorithms. Expert Syst. Appl..

[31] Oreski D., Oreski S., Klicek B. (2017). Effects of dataset characteristics on the performance of feature selection techniques. Appl. Soft Comput..

[32] Xue J., Shen B. (2020). A novel swarm intelligence optimization approach: Sparrow search algorithm. Syst. Sci. Control Eng..

[33] Mirjalili S. (2015). Moth-flame optimization algorithm: A novel nature-inspired heuristic paradigm. Knowl.-Based Syst..

[34] Hashim F.A., Hussien A.G. (2022). Snake Optimizer: A novel meta-heuristic optimization algorithm. Knowl.-Based Syst..

[35] Shadravan S., Naji H.R., Bardsiri V.K. (2019). The Sailfish Optimizer: A novel nature-inspired metaheuristic algorithm for solving constrained engineering optimization problems. Eng. Appl. Artif. Intell..

[36] Cuzick J. (1985). A Wilcoxon-type test for trend. Stat. Med..

[37] Asuncion A., Newman D. (2007). UCI Machine Learning Repository. https://archive.ics.uci.edu/ml/.

[38] Jain I., Jain V.K., Jain R. (2018). Correlation feature selection based improved-binary particle swarm optimization for gene selection and cancer classification. Appl. Soft Comput..

[39] Cover T., Hart P. (1967). Nearest neighbor pattern classification. IEEE Trans. Inf. Theory.

[40] Gao K., Khoshgoftaar T.M., Wang H., Seliya N. (2011). Choosing software metrics for defect prediction: An investigation on feature selection techniques. Softw. Pract. Exp..

